# Toward Enhanced State of Charge Estimation of Lithium-ion Batteries Using Optimized Machine Learning Techniques

**DOI:** 10.1038/s41598-020-61464-7

**Published:** 2020-03-13

**Authors:** M. A. Hannan, M. S. Hossain Lipu, Aini Hussain, Pin Jern Ker, T. M. I. Mahlia, M. Mansor, Afida Ayob, Mohamad H. Saad, Z. Y. Dong

**Affiliations:** 1 Department of Electrical Power Engineering, College of Engineering, Universiti Tenaga Nasional, Kajang, 43000 Malaysia; 2 Centre for Integrated Systems Engineering and Advanced Technologies, FKAB, Universiti Kebagsaan Malaysia, Bangi, 43600 Malaysia; 3 School of Information, Systems and Modelling, University of Technology Sydney, Sydney, Australia; 4 School of Electrical Engineering and Telecommunications, UNSW, Sydney, Australia

**Keywords:** Energy science and technology, Energy harvesting, Energy storage, Renewable energy, Engineering, Electrical and electronic engineering

## Abstract

State of charge (SOC) is a crucial index used in the assessment of electric vehicle (EV) battery storage systems. Thus, SOC estimation of lithium-ion batteries has been widely investigated because of their fast charging, long-life cycle, and high energy density characteristics. However, precise SOC assessment of lithium-ion batteries remains challenging because of their varying characteristics under different working environments. Machine learning techniques have been widely used to design an advanced SOC estimation method without the information of battery chemical reactions, battery models, internal properties, and additional filters. Here, the capacity of optimized machine learning techniques are presented toward enhanced SOC estimation in terms of learning capability, accuracy, generalization performance, and convergence speed. We validate the proposed method through lithium-ion battery experiments, EV drive cycles, temperature, noise, and aging effects. We show that the proposed method outperforms several state-of-the-art approaches in terms of accuracy, adaptability, and robustness under diverse operating conditions.

## Introduction

Lithium battery technologies have increasingly advanced toward the large market of electric vehicles (EVs) because of their high specific power, specific energy, long lifespan, and small size and weight^1^. Researchers worldwide have focused on the development of lithium-ion batteries in terms of material, performance, life cycle, and cost^2^. The issues and concerns on lithium-ion battery charging and discharging control, state of charge (SOC) evaluation, temperature control, fault diagnosis, and battery protection have been extensively investigated^3^. SOC is a significant parameter of lithium-ion batteries and indicates the charge level of a battery cell to drive an EV^4,5^. SOC estimation of lithium-ion batteries is compulsory for the safe and efficient operation of EVs. An accurate SOC estimation method improves the battery lifespan by controlling overcharge and overdischarge states^6^. However, accuracy of SOC is influenced by electrochemical reactions, material degradation, and aging cycles. The existing key issues regarding SOC estimation approaches include inappropriate battery model, complex computation, poor robustness, and slow convergence speed caused by noise and temperature deviations^6,7^. Hence, an enhanced SOC estimation algorithm should be developed to achieve secure and steady operation of lithium-ion battery storage systems.

SOC estimation of lithium-ion batteries is commonly estimated using three methods, namely, conventional^8,9^, model-based^10–12^, and machine learning (ML) approaches^13–15^. Conventional approaches are simple but are unsuitable for online operations^16^. The model-based methods are known as the traditional approaches which can be extremely powerful to model the behavior of lithium-ion batteries accurately^17^. Nevertheless, both practical and theoretical concerns cause difficulty in designing a perfect model for lithium-ion battery SOC estimation. From a practical point of view, the model-based SOC estimation model needs in-depth research, laborious experiments and extended timeframe. On the theoretical side, the model-based SOC estimation methods depend on comprehensive knowledge on battery chemistry, physics and chemical reactions which is composed of many complex mathematical equations, thus leading to complications for battery model development and parameter estimation^18^. On the contrary, the ML-based SOC estimation approaches utilize influx of data and powerful processers to estimate SOC with limited prior knowledge about battery internal characteristics and chemical reactions^19,20^. However, accuracy and performance of the ML methods depend heavily on the quality and amount of the data since unbalanced data would lead to overfitting and underfitting problems^21^.

The scientific innovation of this paper is to introduce an optimized ML technique for SOC evaluation towards the advancement of sustainable EV technologies. ML techniques have received huge attention for their enhanced learning capability, generalization performance, convergence speed, and high accuracy, hence it can be ideal to address the complex and nonlinear characteristics of lithium-ion batteries. However, the hyperparameters selection of ML algorithms by inefficient trial and error leads to computation complexity, such as slow training speed and data fitting problem, thereby delivering unsatisfactory SOC results^22–24^. Currently, the optimization techniques have been increasingly popular to achieve high adaptability, improved efficiency, and high-quality results thus can be employed to determine the optimal hyperparameters as well as appropriate training algorithm, and activation function of ML algorithms. Therefore, a proper combination of ML algorithm and optimization technique not only resolves the computational complexity of ML algorithms but also achieves excellent solutions in lithium-ion battery SOC estimation.

In this study, we present a new method for accurate SOC estimation using an ML-based optimization technique. Recurrent nonlinear autoregressive with exogenous inputs (RNARX) neural network algorithm is a well-known subclass of ML algorithm that has been widely used in designing time-series and dynamic systems. The computational capability of RNARX is enhanced by using lightning search algorithm (LSA), thereby increasing SOC estimation accuracy. The results show that the proposed method is accurate and robust because it can accurately examine SOC under different operating conditions. The key contributions of this study are highlighted below:

- The proposed RNARX-LSA algorithm does not require an added filter in the data pre-processing steps rather only needs sensors to monitor the battery signals such as voltage, current, and temperature.
- The RNARX algorithm updates the learning parameters including weights and bias by self-learning algorithm while using the past and present information of the input layer along with past information of the output layer to examine SOC. In contrast, the model-based SOC estimation is designed based on the deep understanding and knowledge of the lithium-ion battery background processes.
- The RNARX-LSA based SOC estimation method does not require the battery model, thus avoiding time and efforts to construct robust rules and mathematical relationships in capturing the battery behavior as well as estimating battery model parameters.
- The SOC estimation by traditional RNARX algorithm uses inefficient trial and error method to find the optimal values of hyperparameters which leads to data overfitting or under-fitting problems. Thus, the training operation of RNARX could consume substantial time to find the correct values of hyperparameters. Hence, LSA is combined with RNARX algorithm to find the best values of hyperparameters which eventually improves the accuracy of SOC estimation under changing environmental conditions.
- The proposed ML-based SOC estimation is validated by experiments and different EV drive cycles under varying temperatures conditions in order to prove the adaptability and generalization capability. In addition, the accuracy and robustness of the RNARX-LSA model are further verified under different noise effects and aging cycles. The proposed method is suitable for online battery management system (BMS) since the execution of SOC in real-time is extremely fast due to low mathematical complications in the testing stage.

## Results

### SOC estimation through constant discharge test (CDT)

**Figure 1:**
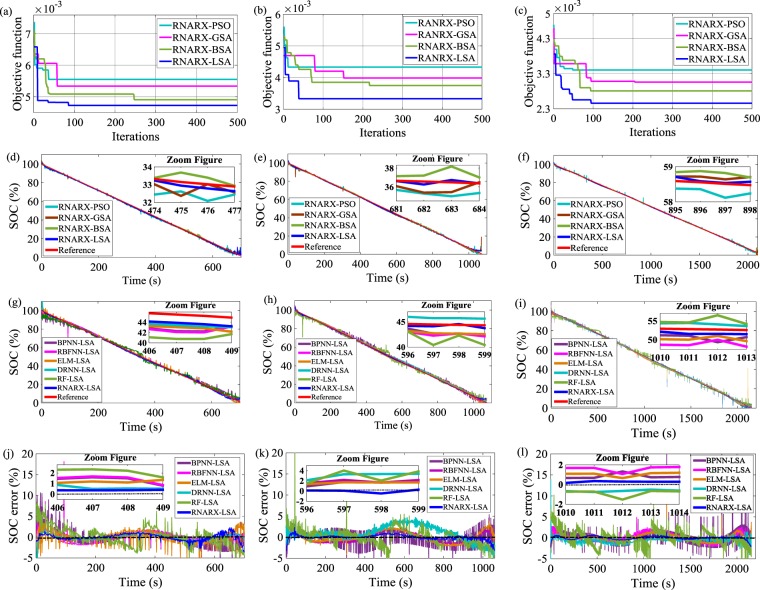
Optimization response curve for CDT load profile (**a**) 1.5 C CDT, (**b**) 1 C CDT, and (**c**) 0.5 C CDT; SOC performance comparison with different optimization methods; (**d**) 1.5 C CDT, (**e**) 1 C CDT, and (**f**) 0.5 C CDT; SOC performance comparison with state-of-the-art ML methods (**g**) 1.5 C CDT (**h**) 1 C CDT, and (**i**) 0.5 C CDT; SOC error estimation (**j**) 1.5 C CDT, (**k**) 1 C CDT, and (**l**) 0.5 C CDT.

The SOC experimental results under different discharge current rates are presented in this section. The superiority of LSA is compared with three powerful optimization algorithms, namely, backtracking search optimization (BSA), gravitational search algorithm (GSA), and particle swarm optimization (PSO) methods. As shown in Fig. 1, LSA performs better compared with BSA, GSA, and PSO algorithms in achieving the minimum objective function and accurate SOC estimation results. The best values of input delays (IDs), feedback delays (FDs), and hidden neurons (HNs) of RNARX are calculated by monitoring the lowest value of the objective function in the optimization response curves. For example, in a 1.5-coulomb (C) constant discharge test (CDT), the minimum value of objective function of 4.72 × 10^−3^ is achieved after 86 iterations which provide the optimal values of IDs, FDs, and HNs of 2, 4, and 7, respectively. Similar procedures are applied in 1 and 0.5 C CDTs to determine the optimal hyperparameters. The SOC estimation results are compared with the reference SOC. The SOC estimated using RNARX-LSA is placed adjacent to the reference SOC value, whereas the RNARX-based BSA, GSA, and PSO deviate from the reference SOC value. For instance, in 1.5 C CDT, root mean square error (RMSE) in the proposed method is estimated to be 0.8937% which is lower than that of RNARX based BSA, GSA, and PSO algorithms. The results are also enhanced in the case of MAE which drops by 48.9%, 38.6%, 36.4% compared with RNARX based BSA, GSA, and PSO algorithms, respectively. The performance of RNARX-LSA for SOC estimation is compared with state-of-the-art ML algorithms, including backpropagation neural network (BPNN), radial basis function NN (RBFNN), extreme learning machine (ELM), deep recurrent NN (DRNN), and random forest (RF) algorithms. These popular ML algorithms are optimized using LSA to perform a fair comparative analysis. It is found that SOC estimation obtained using RNARX-LSA approximately aligns with the reference SOC values while SOC estimation using other optimized ML methods diverge and locate distant from the actual SOC values. In 1 C CDT, RMSE of RNARX-LSA is computed to be 0.6858%, indicating 61%, 44.6%, 49.3%, 38.9%, and 65.5% reductions from BPNN-LSA, RBFNN-LSA, ELM-LSA, DRNN-LSA, and RF-LSA, respectively. In 0.5 C CDT, the mean square error (MSE) is computed to be 0.0014% that declines by 94.1%, 89.8%, 91%, 85.7%, and 95.7% in comparison with BPNN-LSA, RBFNN-LSA, ELM-LSA, DRNN-LSA, and RF-LSA algorithms, respectively. In all test conditions, RNARX-LSA algorithm delivers reasonable accuracy while maintaining the SOC error below ± 5%.

### SOC estimation through hybrid pulse power characterization (HPPC) test

**Figure 2:**
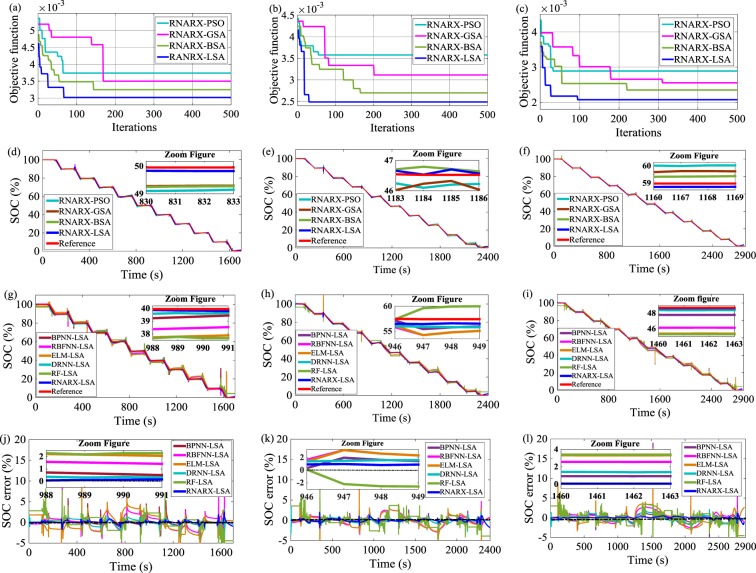
Optimization response curve for constant discharge current load profile (**a**) HPPC 0.25 C discharge current, (**b**) HPPC 0.1 C discharge current, and (**c**) HPPC 0.07 C discharge current; SOC performance comparison with different optimization methods (**d**) HPPC 0.25 C discharge current, (**e**) HPPC 0.1 C discharge current, and (**f**) HPPC 0.07 C discharge current; SOC performance comparison with state-of-the-art ML methods (**g**) HPPC 0.25 C discharge current, (**h**) HPPC 0.1 C discharge current, and (**i**) HPPC 0.07 C discharge current; SOC error estimation (**j**) HPPC 0.25 C discharge current, (**k**) HPPC 0.1 C discharge current, and (**l**) HPPC 0.07 C discharge current.

A comparative study of LSA, BSA, GSA, and PSO is conducted by developing the optimization response curve and associated objective function values, as shown in Fig. 2. The performance of LSA is superior in the HPPC load profile, where LSA achieves the lowest value of the objective function compared with BSA, GSA, and PSO. The lowest value of the objective function is estimated to be 3 × 10^−3^ after 67 iterations in HPPC 0.25 C load profile. Accordingly, IDs, FDs, and HNs related to the said iteration are computed to be 7, 2, and 10, respectively. Similar processes are used in other HPPC load profiles. The LSA results achieved from optimization response curves support the SOC estimation results. The RNARX-LSA-based SOC estimation results are found to be located near to the actual SOC values, whereas the SOC estimation results examined by RNARX-BSA, RNARX-GSA, and RNARX-PSO deviate from the reference SOC values. In HPPC 0.25 C load profile, the RMSE in RNARX-LSA is low and estimated to be 0.4302%, indicating 39.1%, 29.8%, and 19.7% reductions compared with RNARX-BSA, RNARX-GSA, and RNARX-PSO, respectively. Besides, the MAE declines by 57.4%, 50.4% and 43% in comparison with RNARX-BSA, RNARX-GSA, and RNARX-PSO methods, respectively. Similar results are also obtained in HPPC 0.1 C and HPPC 0.07 C load profiles. RNARX-LSA is dominant in other LSA-optimized ML methods. In HPPC 0.25 C load profile, the mean absolute error (MAE) of RNARX-LSA is 0.2287%, indicating 43.5%, 79.8%, 86.9%, 52%, and 83.3% reductions compared with BPNN-LSA, RBFNN-LSA, ELM-LSA, DRNN-LSA, and RF-LSA, respectively. The performance of SOC is also enhanced in obtaining small RMSE, MSE, MAE, mean absolute percentage error (MAPE) and standard deviation (SD) values. In HPPC 0.1 C load profile, the RNARX-LSA has MAPE of 5.8573% which is a reduction of 10.8%, 35.2%, 36.8%, 6.5% and 36.7% in comparison with the BPNN-LSA, RBFNN-LSA, ELM-LSA, DRNN-LSA and RF-LSA methods, respectively. In HPPC 0.07 C load profile, MSE decreases by 84.8%, 95.8%, 97.6%, 56.3% and 97.4% in RNARX-LSA compared with BPNN-LSA, RBFNN-LSA, ELM-LSA, DRNN-LSA, and RF-LSA methods, respectively.

### SOC estimation through dynamic stress test (DST)

**Figure 3:**
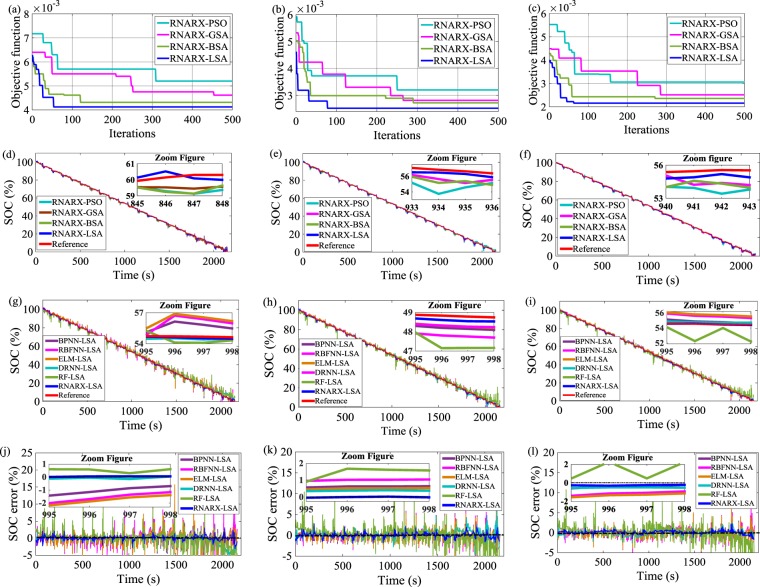
Optimization response curve for DST drive cycle (**a**) 0 °C, (**b**) 25 °C, and (**c**) 45 °C; SOC performance comparison with different optimization techniques (**d**) 0 °C, (**e**) 25 °C, and (**f)** 45 °C; SOC performance comparison with state-of-the-art ML methods (**g**) 0 °C, (**h**) 25 °C, and (**i**) 45 °C; SOC error estimation (**j**) 0 °C, (**k**) 25 °C, and (**l**) 45 °C.

The objective functions in the DST drive cycle under 0 °C, 25 °C, and 45 °C are evaluated from the optimization response curve, as outlined in Fig. 3. LSA achieves excellent performance in obtaining the minimum value of objective function compared with BSA, GSA, and PSO. The results show that the lowest values of objective function achieved by LSA are computed to be 4.12 × 10^−3^, 2.52 × 10^−3^, and 2.15 × 10^−3^ after 55, 78, and 63 iterations at 0 °C, 25 °C, and 45 °C, respectively. Accordingly, the optimal values of IDs, FDs, and HNs of 2, 1, 7; 6, 4, 5, and 3, 2, 15 are obtained at 55, 78, and 63 iterations, respectively. The RNARX-LSA-based SOC estimation method is outstanding in delivering low SOC error, RMSE, and MAE. At 25 °C, the RMSE and MAE of RNARX-LSA are 0.4907% and 0.3449%, respectively, which are lower than RNARX-BSA, RNARX-GSA, and RNARX-PSO. The performance is also compared with LSA-optimized ML methods. For example, at 45 °C, approximately 48.5%, 62.3%, 55.1%, 34.5%, and 74.9% reductions are noted in RNARX-LSA compared with BPNN-LSA, RBFNN-LSA, ELM-LSA, DRNN-LSA, and RF-LSA, respectively, in calculating the RMSE. It is also evident that the SOC error rates decline as the temperature increases due to the rise of the electrolyte activity inside the lithium-ion battery cell. Hence, the capacity of the battery elevates as the temperate accelerates from 0 °C to 45 °C^25^. For instance, the proposed method has RMSE of 0.41% at 45 °C which decreases by 26.4% and 15.5% in comparison with the values obtained at 0 °C and 25 °C, respectively. Similarly, at 45 °C, MSE, MAE, MAPE and SD values reduce by 46.8%, 17.8%, 13.1%, and 27.3% compared with the values derived at 0 °C, respectively.

### SOC estimation through federal urban driving schedule (FUDS)

**Figure 4:**
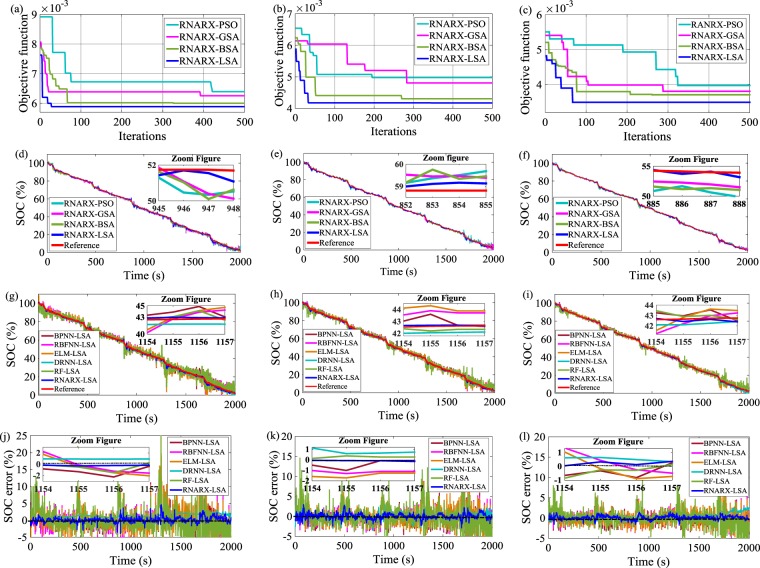
Optimization response curve for FUDS drive cycle (**a**) 0 °C, (**b**) 25 °C, and (**c**) 45 °C; SOC performance comparison with different optimization techniques (**d**) 0 °C, (**e**) 25 °C, and (**f**) 45 °C; SOC performance comparison with state-of-art ML methods (**g**) 0 °C, (**h**) 25 °C, and (**i**) 45 °C; SOC error estimation (**j**) 0 °C, (**k**) 25 °C, and (**l**) 45 °C.

The optimization response curves are generated for the FUDS drive cycle, and the minimum value of objective functions is noted to obtain the best value of hyperparameters of the RNARX algorithm. The optimization performance comparison of LSA, BSA, GSA, and PSO under three different temperatures is displayed in Fig. 4. LSA achieves the lowest value of objective function among all optimization techniques, achieving 5.89 × 10^−3^, 4.17 × 10^−3^, and 3.49 × 10^−3^ after 29, 34, and 67 iterations, respectively. The corresponding iterations deliver the appropriate value of IDs, FDs, and HNs of 4, 1, 15; 2, 2, 18, and 3, 2, 17 at 0 °C, 25 °C, and 45 °C, respectively. The RMSE, and MAE, values ensure the superiority of RNARX-LSA performance compared with RNARX-BSA, RNARX-GSA, and RNARX-LSA. The MAE of RNARX-LSA at 0 °C decreases by 42.9%, 35%, and 13.8% compared with RNARX-BSA, RNARX-GSA, and RNARX-PSO, respectively. RNARX-LSA has lower RMSE, MSE, MAE, MAPE, SD values than that of BPNN-LSA, RBFNN-LSA, ELM-LSA, DRNN-LSA, and RF-LSA. At 25 °C, the RMSE of the proposed method decreases by 89.4%, 92%, 91.7%, 62.6%, and 91.7% compared with BPNN-LSA, RBFNN-LSA, ELM-LSA, DRNN-LSA, and RF-LSA, respectively. Similarly, at 25 °C, MAPE declines by 58.5%, 62%, 53.2%, 41.7% and 51.8% in comparison with BPNN-LSA, RBFNN-LSA, ELM-LSA, DRNN-LSA, and RF-LSA, approaches respectively. It is also reported that the change in temperature affects the SOC estimation results. For example, at 0 °C, MAE is achieved to be 0.58% in the proposed method which is a rise of 15.4% and 54.8% form the value obtained at 25 °C and 45 °C, respectively. Likewise, at 25 °C, RMSE, MSE, MAPE, and SD values increase by 38.9%, 50.2%, 72.1%, and 39.6%, compared with the values derived at 45 °C, respectively. The SOC error is also found narrow and remained under ±5%, ±4%, and ±2% at 0 °C, 25 °C, and 45 °C, respectively.

### SOC robustness against noise effects

**Figure 5:**
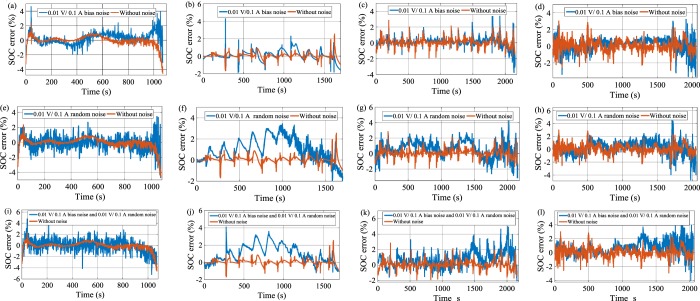
SOC error estimation results under 0.01 V/0.1 A bias noise (**a**) 1 C CDT, (**b**) HPPC 0.25 C discharge current, (**c**) DST, and (**d**) FUDS; SOC error estimation results under 0.01 V/0.1 A random noise (**e**) 1 C CDT, (**f**) HPPC 0.25 C discharge current, (**g**) DST, and (**h**) FUDS; SOC error estimation results under 0.01 V/0.1 A bias noise and 0.01 V/0.1 A random noise (**i**) 1 C CDT, (**j**) HPPC 0.25 C discharge current, (**k**) DST, and (**l**) FUDS.

The SOC performance is evaluated against bias noise through experimental tests and EV drive cycles, as shown in Fig. 5. The results show that the RMSE and maximum SOC error in HPPC 0.25 C discharge load profile are computed to be 0.5885% and 4.33%, respectively. The results are reasonable in 1 C CDT, where the RMSE and maximum SOC error are found to be 0.8404% and 4.67%, respectively. The addition of bias noise to EV drive cycles does not deviate the SOC estimation results considerably, where the proposed approach achieves RMSE and maximum SOC error values of 0.8086%, and 3.42%, respectively in DST drive cycle. Likewise, in FUDS drive cycle, RMSE and maximum SOC error values are obtained to be 0.7865% and 3.25%, respectively. The SOC estimation results are satisfactory versus random noise when limiting the SOC error range of ± 5%. The maximum SOC error is under 4% in 1 C CDT and HPPC 0.25 C load profiles. Besides, RMSE is calculated to be 3.47% and 3.51% in 1 C CDT and HPPC 0.25 C load profiles, respectively. The results are suitable in the case of the EV drive cycles, where the maximum SOC error is less than ± 5%. The RMSE in DST and FUDS drive cycles is estimated to be 1.1373%, and 1.0268% respectively. Accordingly, the maximum SOC error is achieved to be 4.88%, and 4.55% in DST and FUDS drive cycles. The SOC estimation results are verified through the combination of bias and random noises. The results indicate that the mixture of bias and random noises has a small impact on SOC estimation in terms of SOC error and RMSE. The maximum SOC error is slightly higher than in the two previous cases although the error remains inside the acceptable range of ±5%. The maximum SOC errors of 4.82% and 4.13% are obtained in 1 C CDT and HPPC 0.25 C load profiles, respectively. Accordingly, the RMSE values are calculated to be 1.1569% and 1.4221%, respectively. The results are satisfactory under EV drive cycles, where the RMSE is 1.2061%, and 1.1306% in DST and FUDS drive cycles, respectively. Consequently, the maximum SOC error is limited to 4.98%, and 4.87% in DST and FUDS drive cycles, respectively. The RNARX-LSA-based SOC estimation method exhibits strong robustness against biased and random noises.

### SOC evaluation under aging effects

**Figure 6:**
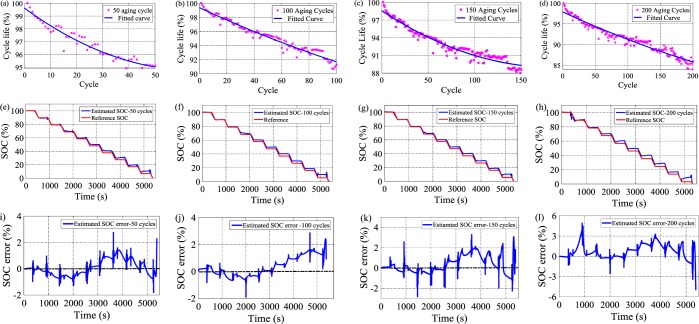
Battery cycle life profile of LiNCA battery under different aging cycles (**a**) 50 aging cycle, (**b**) 100 aging cycle, (**c**) 150 aging cycle, and (**d**) 200 aging cycle; SOC performance under (**e**) 50 aging cycle, (**f**) 100 aging cycle, (**g**) 150 aging cycle, and (**h**) 200 aging cycle; SOC error results under (**i**) 50 aging cycle, (**j**) 100 aging cycle, (**k**) 150 aging cycle, and (**l**) 200 aging cycle.

The proposed method achieves excellent SOC estimation results for a fresh lithium-ion battery. The accuracy of the lithium-ion battery decreases after the battery is cycled for hundreds of times. Hence, the accuracy and robustness of the proposed method are evaluated under different aging cycles. The lithium-ion battery degradation performance is evaluated under four milestone aging cycles, namely, 50, 100, 150, and 200 cycles, as shown in Fig. 6. The cycle life of LiNiCoAlO_2_ (LiNCA) battery is obtained to be 85.92% after 200 aging cycles, which reduces by 9.6% compared with the value achieved after 50 aging cycles. Likewise, the capacity is found to be 3052 mAh after 50 aging cycles and reduces to 2763 mAh after 200 aging cycles. RNARX-LSA is trained using the HPPC experimental dataset of a new LiNCA battery, whereas the dataset of aged LiNCA battery for 50, 100, 150, and 200 cycles is used to test the performance of the trained model. The proposed method achieves RMSE, MSE, MAE, MAPE, SD and SOC error of 0.59%, 0.0036%, 0.48%, 2.98%, 0.57% and [−1.84%, 2.78%], respectively, under 50 aging cycles. The SOC accuracy drops at 100 aging cycles with RMSE, MSE, MAE, MAPE, SD and SOC error of 0.78%, 0.0063%, 0.61%, 3.25%, 0.78% and [−1.92%, 2.89%], respectively. The SOC accuracy further declines when the battery is deeply cycled. For instance, RMSE, MSE, MAE, MAPE, SD and SOC error are achieved to be 1.2%, 0.016%, 0.96%, 4.61%, 1.25% and [−4.84%, 4.91%], respectively under 200 aging cycles. However, in all aging cycle conditions, SOC error stays below ± 5%.

### Comparative validation with the existing methods

The accuracy and robustness of RNARX-LSA method are further investigated by evaluating different SOC error rate terms as depicted in Table 1. The recent and notable studies concerning both traditional and ML-based SOC estimation methods are considered for comparative analysis. The most influential factors related to SOC estimation such as lithium-ion battery type, temperature, load profile are employed to analyze the results. It is observed that RNARX-LSA based SOC estimation method outperforms the existing SOC estimation approaches under different EV drive cycles. For instance, RMSE is estimated to be over 1% in BPNN, ELM, CNN, LSTM, GRU and GFCA methods whereas RMSE is found under 1% in the proposed approach. Apart from ML techniques, the error rates are also high in conventional methods and model-based approaches with RMSE over 1% in OCV, UPF, RLS, and PIO methods. Moreover, MAE is estimated below 0.6% in the proposed method while that for RBFNN, DNN, WNN, and GPR is above 0.7%. The proposed model is also dominant in other SOC estimation techniques under CDT and HPPC environments. For example, the proposed method computes MAE of 0.6858% in 1 C CDT; however, MAE is reported over 2% in UKF and H∞ Filter methods. Besides, NLO has RMSE of 1.49% in HPPC load profile whereas the proposed algorithm achieves RMSE of 0.4302%. All the results shown above indicate that the proposed method is accurate, robust and superior to the existing popular SOC estimation approaches under different operating conditions.

**Table 1:** Performance comparison between the proposed method and the existing methods.

Method	Battery type	Battery capacity	Temperature	Validation profiles	Error rate
OCV^56^	LiFePO_4_	1.1 Ah	0 °C to 50 °C at an interval of 10 °C	DST, FUDS	RMSE 5%
CC^57^	Lithium-ion cell	2.3 Ah	Room temperature	C-rates Charging-discharging current	MAE 1.905%
UKF^45^	LiNMC	24 Ah	Room temperature at 25 ° C ± 2 ° C	1 C CDT	MAE 2.56%
					Max SOC error 5.36%
H∞ Filter^58^	Lithium-ion cell	2.4 Ah	Constant temperature	1 C CDT	MAE 3.96%
UPF^59^	10 Ah LiFePO_4_	10 Ah	−20 °C~50 °C	EV operation condition	RMSE 2.05%
RLS^60^	90 Ah LiFePO_4_	90 Ah	−10 °C~50 °C	Urban EV drive cycle	RMSE 2.3%
					MAE 1.8%
SMO^61^	Lithium polymer battery	5 Ah	Room temperature	1 C CDT	RMSE 1.8%
PIO^8^	Lithium-ion cells	90 Ah	0 °C, 25 ° C, 40 °C	DST	RMSE 1.2%
NLO^47^	lithium-ion battery	10 Ah	Room temperature	HPPC	RMSE 1.49%
BPNN^14^	LiNiMnCoO_2_/NMC	2 Ah	0 °C, 25 °C and 45 °C	DST and FUDS	RMSE 0.48%~1.47% in DST
					RMSE 0.57~1.74% in FUDS
RBFNN^54^	LiMn_2_O_4_	6 Ah	0 °C, 25 °C, and 40 °C	UDDS, ECE	MAE < 5%
ELM^6^	LiNiMnCoO_2_/NMC	2 Ah	0 °C, 25 °C and 45 °C	DST, FUD and US06	RMSE of 1.1% in DST
					RMSE 1.4% in FUDS
					RMSE 1.8% in US06
DNN^13^	Panasonic LiNiCoAlO_2_/NCA	2.9 Ah	0 °C, 10 °C, and 25 °C	US06 and HWFET	MAE 1.35% in HWFET
					MAE 1.85% in US06
LSTM^20^	Panasonic LiNiCoAlO_2_/NCA	2.9 Ah	0 °C, 10 °C and 25 °C	Dynamic drive cycles, ±18 A,	RMSE 1.11% ~2.44%
					MAE 0.77~2.08%
CNN^23^	LiNMC	1.3 Ah	0 °C to 50 °C at an interval of 10 °C	FUDS	RMSE 1.3~4.5%
					MAE 0.95~3.04%
GRU^62^	LiFePO_4_	2.3 Ah	0 °C, 30 °C and 50 °C	DST and FUDS	RMSE 0.64~1.97% in DST
					RMSE 0.83~2.45% in FUDS
					MAE 0.5~1.44% in DST
					MAE 0.49~1.77% in FUDS
ANFIS^63^	36 Lithium-Ion cells	40 Ah	Temperature range from −30 °C to 55 °C	Charging and discharging currents (i) 57 A, (ii) 68 A	MSE 3.4% in 57 A
					MSE 5.6% in 68 A
WNN^51^	Samsung ICR-18685-22P	2150 mAh	Room temperature	NEDC and UDDS	MAE 0.72% in NEDC
					MAE 0.71% in UDDS
GFCA^22^	lithium-ion cell	100 Ah	24 °C ± 2 °C	FUDS	RMSE 1.68%
GPR^64^	LiNiMnCoO_2_/NMC	2 Ah	Room temperature	US06	MAE 0.8119%
Proposed method	LiNCA for CDT and HPPC tests	3.2 Ah	Room temperature	CDT and HPPC	RMSE 0.6858% for 1 C CDT
					RMSE 0.4302% for HPPC 0. 25 C discharge
	LiNMC For EV drive cycles	2 Ah	0 °C, 25 °C, and 40 °C	DST and FUDS	RMSE 0.4174~0.5637% in DST
					RMSE 0.4906~0.8694% in FUDS
					MAE 0.3164~0.3847% in DST
					MAE 0.3797~0.5881% in FUDS

OCV: Open Circuit Voltage, CC: Coulomb Counting, UKF: Unscented Kalman Filter, UPF: Unscented Particle Filter, RLS: Recursive least Square, SMO: Sliding Mode Observer, PIO: Proportional Integral Observer, NLO: Non-linear Observer, RNN: Recurrent Neural Network, DNN: Deep Neural Network, LSTM: Long Short Term Memory Network, CNN: Convolutional Neural network, GRU: Gated Recurrent Unit, ANFIS: Adaptive Neuro-Fuzzy Inference System, WNN: Wavelet Neural Network, GFCA: Genetic Fuzzy Clustering Algorithm, GPR: Gaussian Process Regression, UDDS: Urban Dynamometer Driving Schedule; ECE: Economic Commission of Europe, RNN: Recurrent Neural Network, HWFET: Highway Fuel Economy Test, NEDC: New European Driving Cycle, UDDS: Urban Dynamometer Driving Schedule.

## Discussion

In this article, we validate RNARX-LSA for SOC estimation using the experimental data obtained through CDT and HPPC tests. We use different discharge current rates to evaluate the accuracy of the proposed model. An extensive comparative study between LSA and BSA, GSA, and PSO is performed through the assessment of objective function using the same iterations and population size. The proposed RNARX-LSA provides better results than that of RNARX-BSA, RNARX-GSA, and RNARX-PSO in obtaining the lowest objective function and small SOC error under CDT and HPPC tests. The robustness, adaptability, and efficiency of the proposed model are examined under DST and FUDS EV drive cycles. SOC is evaluated under three different temperatures, namely, 0 °C, 25 °C, and 45 °C. The RNARX-LSA-based SOC estimation method achieves excellent results and delivers minimum SOC error compared with RNARX-BSA, RNARX-GSA, and RNARX-PSO under different EV drive cycles and temperature conditions. The proposed method exhibits better outcomes than that of state-of-the-art optimized ML methods in terms of reducing RMSE and MAE. The robustness of the proposed model is assessed against biased and random noises. The SOC performance is verified under four milestone aging cycles, namely, 50, 100, 150, and 200 cycles. In all test conditions, the developed method achieves satisfactory results. We conclude that RNARX-LSA is demonstrated as a generalized model that can accurately assess the SOC under different operating conditions.

## Methods

### SOC equation

SOC is calculated by assessing the current capacity divided by the nominal capacity, which is expressed in the following equation^7^:

1 $$SOC=SO{C}_{0}-\frac{1}{{C}_{n}}\int i.\eta dt$$

where *SOC* is the estimated value, $SO{C}_{0}$ is the reference value, ${C}_{n}$ is the nominal capacity, $\eta$ is the coulombic efficiency, and *i* and *t* denote the battery charging/discharging current and duration, respectively.

### Experiments and data development

A test bench model was established with lithium-ion battery batteries for data extraction and SOC evaluation. The test bench is divided into two parts, namely, hardware and software parts. The hardware part comprises LiNCA batteries and a NEWARE battery testing system (BTS)−4000. LiNCA has a rated capacity, nominal voltage, and cut-off voltage of 3200 mAh, 3.6 V, and 2.5 V, respectively. The software part is designed using MATLAB 2015a and a software version 7.6 related to BTS-4000. A host computer was used to collect data from hardware and install the software. The BTS-4000 measurement unit was connected to a NEWARE BTS-4000 control unit through the RS485 port, whereas the control unit was connected to a host computer through a TCP/IP port. The steps of CDT and HPPC tests were executed using the necessary software actions of BTS software. BTS software was used to conduct the battery experimental test at the different charge and discharge current rates. The charging and discharging control of LiNCA battery was operated using the appropriate function of BTS software version 7.6 while satisfying the cut-off current and voltage values instructed by the manufacturer. The experimental dataset, including current and voltage, was recorded in each second and kept in the database storage system of the host computer. Subsequently, the dataset was transferred to MATLAB 2015a software to execute RNARX-LSA algorithm.

### Training and testing dataset

The entire dataset was divided into two subsets, namely, training and testing subsets. Cross-validation was applied to randomly split the data into training and testing at 70:30 ratio. The efficiency and robustness of the training data of RNARX-LSA can be enhanced through appropriate data normalization. Data normalization can enhance the convergence rate and remove the negative influence. In this study, the input dataset was normalized to a range [−1, 1], as expressed in the following equation^26^,

2 $$x{\prime} =\frac{2(x-{x}_{min})}{{x}_{max}-{x}_{min}}-1$$

where ${x}_{max}$ is the maximum value, and ${x}_{min}$ is the minimum value of input vector $x$. In this study, the performance goal and the number of epochs were set to 0.000001 and 1000, respectively. The host computer was configured with Core i5 2.3 GHz processor and 12 GB RAM to execute the algorithm.

### Objective function formulation

The objective function aims to determine the optimal value of hyperparameters of RNARX algorithm through an iterative process which leads to minimum SOC error rates estimation. In this study, RMSE was chosen as the objective function because of the large number of sample variables and randomness behavior of SOC errors^27^. The objective function is formulated using the following equation^28^,

3 $$RMSE=\sqrt{\frac{1}{n}\mathop{\sum }\limits_{i=1}^{n}{(SO{C}_{{a}_{i}}-SO{C}_{e{s}_{i}})}^{2}}$$

4 $$Objective\,Function=min\,(RMSE)$$

### Optimization constraints

LSA must satisfy the constraints in searching for optimal values and estimating SOC accurately. In this study, the constraints were related to the minimum and maximum ranges of hyperparameters of RNARX algorithm, including IDs, FDs, and HNs. The new updated population of hyperparameters was repeatedly assessed during the iterative process whether they were outside of the boundary region. Otherwise, the outcome of LSA optimization could deviate, thereby delivering poor SOC estimation results. For example, variable ${X}_{i,j}^{k}$ should be between ${X}_{i,j}^{k-1}$ and ${X}_{i,j}^{k+1}$. The hyperparameters of RNARX algorithm should be reproduced with the boundary, and the results will be updated accordingly when variable ${X}_{i,j}^{k}\,\,$is greater than $\,{X}_{i,j}^{k+1}$ or less than ${X}_{i,j}^{k-1}$. Therefore, the appropriate limit of the hyperparameters of RNARX algorithm can be expressed follows:

5 $${X}_{i,j}^{k-1} < {X}_{i,j}^{k} < {X}_{i,j}^{k+1}$$

### Enhanced ML technique

RNN is a supervised ML method designed using three layers, namely, input, hidden, and output layers^29^. RNARX is a prominent subgroup of RNN that uses one or more feedback loops to address complex and time-series problems^30^. SOC estimation of RNARX is performed using the present and past values of inputs and estimated past values of outputs. The output of RNARX can be represented as^31^:

6 $$\begin{array}{c}y(n+1)=\\ {f}_{0}[{b}_{0}+\mathop{\sum }\limits_{h=1}^{N}{w}_{ho}.{f}_{h}({b}_{h}+\mathop{\sum }\limits_{i1=0}^{{d}_{u1}}{w}_{i1h}{u}_{1}(n-i1)+\mathop{\sum }\limits_{i2=0}^{{d}_{u2}}{w}_{i2h}{u}_{2}(n-i2)+\mathop{\sum }\limits_{j=0}^{{d}_{y}}{w}_{jh}y(n-j))]\end{array}$$

where ${b}_{0}$ and ${b}_{h}$ are the biases, ${w}_{ih}$, ${w}_{ho}$, and ${w}_{jh}$ are the weights, and ${f}_{h}$(.) and ${f}_{0}$(.) are the activation functions. ${u}_{1}\,\,$and ${u}_{2}$ denote the first and second inputs, respectively, and $y$ represents the output. The hidden layer and output layer operations are executed using *logsig* and *purelin* transfer functions, respectively^32^.

### Hyperparameter tuning

LSA^33^ is used to find the optimal hyperparameters of the RNARX algorithm that induces IDs, FDs, and HNs. LSA uses three particles known as projectiles, such as transition, space, and lead projectiles, to search for optimal solutions. Transition projectiles create the first-step leader population, *N*, space projectiles attempt to reach the best leader position, and lead projectile represents the best position among *N* numbers of step leaders. Probability density function $f({x}^{T})$ of the transition projectile can be expressed as^34,35^,

7 $$f({x}^{T})=\{\begin{array}{ll}\frac{1}{b-a} & for\,a\le {x}^{T}\le b\\ 0 & for\,x < a\,or\,{x}^{T}\, > b\end{array}$$

where ${x}^{T}$ is a random value, and *a* and *b* represent the lower and upper bounds of the projectile, respectively. The position of space projectile ${P}^{S}=[{p}_{1}^{S},{p}_{2}^{S},{p}_{3}^{S}\ldots \ldots \ldots \ldots .,{p}_{N}^{S}]$ at $step+1$ can be designed in the form of exponential distribution with shaping parameter *µ*. Probability density function $f({x}^{S})$ of a space projectile can be expressed as^34,35^,

8 $$f({x}^{S})=\{\begin{array}{ll}\frac{1}{\mu }{e}^{\frac{-{x}^{S}}{\mu }} & for\,{x}^{S}\ge 0\\ 0 & for\,{x}^{S}\le 0\end{array}$$

The revised position of ${p}_{i}^{S}$ at $step+1$ is represented as^34,35^,

9 $${p}_{i\_new}^{S}={p}_{i}^{S}\pm exprand({\mu }_{i})$$

where $exprand$ represents the exponential random number. The corresponding stepped leader $s{l}_{i}$ moves toward a new position, $s{l}_{i\_new}$, when ${p}_{i\_new}^{S}$ obtains a satisfactory solution at $step+1$ and the capacity of a projectile ${E}_{p\_i}^{S}$ is greater than the energy of step leader ${E}_{sl\_i}$. Otherwise, they remain unmoved until the next step is obtained. The normal probability density function of lead projectile $f({x}^{L})$ is demonstrated using the following equation^34,35^,

10 $$f({x}^{L})=\frac{1}{\sigma \sqrt{2\pi }}{e}^{\frac{-{({x}^{L}-\mu )}^{2}}{2{\sigma }^{2}}}$$

The revised location of ${p}^{L}$ at $step+1$ can be represented as^36,37^,

11 $${p}_{i\_new}^{L}={P}_{i}^{L}+normrand({\mu }_{L},\,{\sigma }_{L})$$

where $normrand$ denotes a random number. Similarly, ${P}_{i}^{L}$ is updated to ${P}_{i\_new}^{L}$ when it achieves a good result at $step+1$ and ${E}_{p\_i}^{L} > {E}_{sl\_i}^{L}$.

LSA was compared with BSA^14^, GSA^6^, and PSO^38^ using the same population size (50) and iteration numbers (500) to ensure a fair assessment. In LSA, channel time was counted as 10. In GSA, gravitational constant *G*_0_ and acceleration *α* were set 100 and 20, respectively. In PSO, acceleration coefficients *c*_1_, *c*_2_, and weight factor *w* were assigned to 2 and 0.5, respectively. The hyperparameters of BPNN^39^, RBFNN^40^, ELM^41^, DRNN^13^, and RF^42^ algorithms were optimized using LSA to conduct a fair comparative analysis. In the BPNN algorithm, LSA was used to find the optimal number of HNs and learning rates. In the RBFNN algorithm, the number of neurons, spread, and width values was optimized using LSA. The optimal number of neurons was obtained using LSA in the ELM algorithm. For DRNN, the number of hidden layers and HNs was optimized using LSA. The best values of trees and leaves were achieved using LSA in the RF algorithm.

### SOC effectiveness measures

The performance of RNARX-LSA-based SOC estimation was verified using different error rate terms. The mathematical equations of these statistical errors are expressed as follows^36,43,44^:

12 $$SOC\,error=SO{C}_{a}-SO{C}_{es}$$

13 $$MSE=\frac{1}{n}\mathop{\sum }\limits_{i=1}^{n}{(SO{C}_{{a}_{i}}-SO{C}_{e{s}_{i}})}^{2}$$

14 $$MAE=\frac{1}{n}\mathop{\sum }\limits_{i=1}^{n}\,(SO{C}_{{a}_{i}}-SO{C}_{e{s}_{i}})$$

15 $$MAPE=\frac{1}{n}\mathop{\sum }\limits_{i=1}^{n}|\frac{SO{C}_{{a}_{i}}-SO{C}_{e{s}_{i}}}{SO{C}_{{a}_{i}}}|$$

16 $$SD=\sqrt{\frac{1}{n-1}\mathop{\sum }\limits_{i=1}^{n}\,{(SO{C}_{error}-\overline{SO{C}_{error}})}^{2}}$$

where $SO{C}_{a}$ is the reference value, $SO{C}_{es}$ is the estimated value, $\overline{SO{C}_{error}}$ is the average value of SOC error and *n* is the number of data observations. The reference SOC is obtained using (1).

### Implementation of RNARX-LSA based SOC estimation algorithm

**Figure 7:**
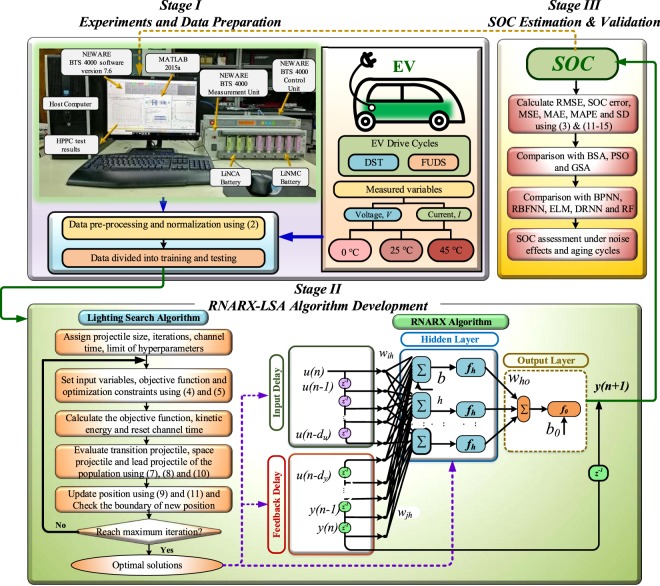
Proposed RNARX-LSA based SOC estimation method structure, execution and validation process.

The execution of the RNARX-LSA algorithm for SOC estimation started with the measurement of battery data including current and voltage from CDT and HPPC experimental tests. After, IDs, FDs, and HNs of RNARX were optimized through the LSA method based on the minimum value of the objective function. The proposed SOC estimation model was then processed into various validation tests to check the model accuracy and robustness under different operating conditions. The SOC estimation results were evaluated using different error rate terms and compared with different optimization techniques and ML approaches. The methodological framework of the proposed RANRX-LSA is illustrated in Fig. 7. The overall implementation procedures are categorized into three stages.

In stage I, the CDT and HPPC battery experimental tests were carried out by developing a test bench model. After, the corresponding dataset was generated including current and voltage from the test bench platform. At the same time, the EV dataset including current, voltage, and temperature was also collected. Then, the data were pre-processed and normalized in order to improve the training speed. Finally, the data partition was performed for algorithm training and testing.

In stage II, the LSA started with assigning the parameters such as population size, iteration number, dimension, input variables, objective function, and optimization constraints. Then, the position of step leader was generated randomly and the objective function was evaluated. After, the channel time was reset by eliminating the bad channel from worst to best. Next, space projectile and lead projectile were ejected and their positions were verified based on the objective function. Subsequently, the location of the projectile was updated if the energy of the projectile was higher than the step leader. After, the population of hyperparameters was reinitialized within the boundary limit. The process continued until it reached the maximum iteration. Finally, the optimal values of hyperparameters were sent to RANRX algorithm and accordingly RNARX training operation was executed using the Levenberg-Marquardt (LM) algorithm and RANRX activation function.

In stage III, SOC was estimated and results were verified using different performance indicators such as RMSE, MSE, MAE, MAPE, SD, and SOC error. Subsequently, a comprehensive comparative analysis was performed with well-known optimization approaches and machine learning methods. Finally, the robustness of SOC was assessed under different temperatures, noise effects and aging cycles.

Figure 1 methods. The CDT experiment^45,46^ started with the charging of LiNCA battery completely using constant current constant voltage (CC-CV) method. A CC of 1.6 A (0.5 C) current was applied until the charge voltage reached 4.2 V. Then, a CV of 4.2 V was employed until the charge current dropped to 0.064 A (0.02 C). Subsequently, the battery was kept idle for 1 h. Next, the discharged current of 1.5 C/1 C /0.5 C was operated until the discharge voltage declined to 2.5 V. The test ended when the battery voltage reached 2.5 V. Otherwise, the battery was discharged again at 1.5 C/1 C /0.5 C.

Figure 2 methods. The HPPC test^47,48^ was executed by generating a combination of charge and discharge current pulses in an orderly manner. The customized HPPC was designed using different charge and discharge current values to verify the robustness of the proposed method. Initially, the battery was charged using CC method with 1.6 A (0.5 C) current until the charge voltage reached 4.2 V. Then, the battery was charged using CV method with 4.2 V until the charge current dropped to 0.064 A (0.02 C). Subsequently, the battery was discharged at 0.5 C/0.3 C/0.1 C for 10 s followed by a rest period of 3 min. Next, the battery was charged at 0.5 C/0.3 C/0.1 C for 10 s followed by a rest period of 3 min. After, the battery was discharged at 0.25 C/0.1 C/0.07 C for 24/60/86 min to decrease the SOC by 10%. The test ended when the battery reached 2.5 V. Otherwise, the battery was discharged again at 0.5 C/0.3 C/0.1 C.

Figures 3 and 4 methods. EV drive cycle data were collected from the Center for Advanced Life Cycle Engineering (CALCE)^49^ battery research group. An 18650 NMC cathode-based lithium-ion battery cell with a nominal capacity of 2.0 Ah and a voltage of 3.6 V was used for SOC estimation. Two different patterns of EV drive cycles, namely, DST and FUDS, were utilized to evaluate SOC performance, as depicted in Figs. 4 and 5, respectively. These drive cycles have diverse current profile in terms of different amplitudes and time durations. The duration of one cycle for DST and FUDS is 360 and 1372 s, respectively^50^. DST corresponds to dynamic charging and discharging, whereas FUDS is related to urban driving. A thermal chamber was used to control the battery temperature. The experiments were conducted at three different temperatures of 0 °C, 25 °C, and 45 °C.

Figure 5 methods. An EV is designed using many sensors and power converters. Electromagnetic interference (EMI) noises are generated when the power converter switching is operated at high frequency, which may add to the measured current and voltage values. Each sensor of EV experiences equipment errors, thereby resulting in error of measured current and voltage signals. Therefore, SOC should be examined against bias and random noises, where bias noise corresponds to the sensor precision, and random noise is related to EMI noises. The robustness of the proposed method was checked under positive bias noises by injecting 0.1 A and 0.01 V to the current and voltage measurements, respectively^51^. In addition to biased noises, a standard random noise with an amplitude of 0.1 A and 0.01 V was added to current and voltage measurements^52^.

Figure 6 methods. Battery aging is important to determine the battery performance after certain aging cycles. The battery capacity decreases with the increase in aging cycles. Firstly, cycle life of LiNCA battery was monitored under different aging cycles. The cycle life was calculated using the current capacity of an aged LiNCA battery cell divided by the capacity of a fresh LiNCA battery cell^53^. The aging operations of LiNCA battery initiated with CC-CV method. The battery was charged until it reached 4.2 V with a current of 1.6 A (0.5 C). Subsequently, the current reduced to 0.064 A (0.02 C), whereas 4.2 V remained constant. The battery was discharged at 1 C (3.2 A) current until the battery voltage reached 2.5 V. One aging schedule was completed when the battery reached 2.5 V. After completion of one aging cycle, the battery was rested for 1 h^54,55^. The process continued for 50, 100, 150, and 200 cycles.

## Supplementary information

Supplementary Information.
