# Supplementary material for: Toward Enhanced State of Charge Estimation of Lithium-ion Batteries Using Optimized Machine Learning Techniques
Source: Sci Rep. 2020 Mar 13;10:4687. doi: 10.1038/s41598-020-61464-7 (PMC7070070; doi:10.1038/s41598-020-61464-7)
Supplement: Supplementary file 1 — Supplementary Information. [file 41598_2020_61464_MOESM1_ESM.pdf]

Supplementary Material

Optimization Algorithms for Machine Learning

M. A. Hannan[1*], M. S. Hossain Lipu[2*], Aini Hussain[2], Pin Jern Ker[1], T. M. I. Mahlia[3], M. Mansor[1], Afida Ayob[2], Mohamad H. Saad[2], Z. Y. Dong[4]

[1]Department of Electrical Power Engineering, College of Engineering, Universiti Tenaga Nasional, Kajang 43000, Malaysia.
[2]Centre for Integrated Systems Engineering and Advanced Technologies, FKAB, Universiti Kebangsaan Malaysia, Bangi 43600, Malaysia.
[3]School of Information, Systems and Modelling, University of Technology Sydney, Australia.
[4]School of Electrical Engineering and Telecommunications, UNSW, Sydney, Australia.
*Corresponding author email (M. A. Hannan): hannan@uniten.edu.my; M. S. Hossain Lipu (lipu@ukm.edu.my)

Backtracking Search Algorithm

Backtracking search algorithm (BSA) is a newly developed meta-heuristic population-based algorithm to solve the complex and non-linear optimization problems. BSA is capable to operate large dimensional problem for the optimum solutions using the historical population and map matrix concepts. With the help of historical population, BSA explores and exploits the better solution to address the local minima trap. On the contrary, map matrix does the necessary correction to change the search direction in order to confirm accurate movement during the exploitation search. BSA is easy to implement with few parameters that are fast, efficient and robust to control parameters. The execution process of BSA is conducted through a gradual process involving some steps namely Selection I, mutation, crossover and Selection II. The detail process of BSA is explained as follows[1],

Step 1: Initialization
The BSA is initialized by generating the numerical values of the initial population and historical population from the uniform distribution of random numbers within the boundary constraints. Also, in this stage, the objective function is being calculated.

$$Primary\ population, Pop_{n,d} \sim \cup\ (low_d, up_d) \quad (1)$$

The representation of the objective function of the primary population,

$$y_{pop} = f(Pop) \quad (2)$$

The historical population is represented as such,

$$HisPop \sim \cup\ (low_d, up_d) \quad (3)$$

The objective function of the historical population,

$$Y_{HisPop} = f(HisPop) \quad (4)$$

where, $x \in \{1,2,3, \dots, N\}$ and $d \in \{1,2,3, \dots, D\}$. Here, the uniform distribution is $\cup$. Each individuals of the primary and historical population is denoted as $Pop_{n,d}$ and $HisPop_{n,d}$ respectively. Y_{pop} represents the objective

function of the total population size where low_d and up_d are the lower and upper limits of the dimension, respectively.

Step 2: Selection-I

The historical population is updated through the iteration process where the 'if-then rule' is utilized. The equation below represents the process of generating the historical population.

$$if a < b, then\ HisPop := Pop | a, b \sim \cup\ (0,1) \tag{5}$$

where, $:=$ represents the 'update' operation.

In BSA algorithm, the population is chosen through random selection from the prior generation of the historical population. The historical population is saved until it is updated. After that, a random shuffling function is applied for revising the order of the individuals.

$$HisPop \coloneqq permuting(HisPop) \tag{6}$$

Step 3: Mutation

In this stage, mutants are generated through the mutation process which involves a process of generating an initial trial population. The formation of the initial trial population is expressed as follows,

$$Mutant = Pop + F.\,(HisPop - Pop) \tag{7}$$

where, F denotes the control parameter, which is used to change the amplitude of the search direction matrix and can be represented by a mathematical representation as follows.

$$F = \ 3.randn \tag{8}$$

where $randn$ is a function which generates normal distribution number $(0{\sim}1)$.

Step 4: Crossover

In this stage, the final trial population is generated through the mutation process within the boundary condition. Typically, this stage comprises two steps as follows.

Step 4(a): Part-I

Firstly, a map matrix comprising the same size of *Pop* is generated in order to find the mutants which are involved in the mutation process. Thus, the target of the *map* matrix is to find the mutants through governing the individuals which are updated through the process of mutation. The equations (9)-(12) below represent the *map* matrix generation process.

$$Map\ matrix\ initialization, map_{(1:N,1:D)} = 1 \tag{9}$$

$$If\ a < b|\ a, b \sim \cup\ (0,1), then$$

$$for\ n\ from\ 1\ to\ N\ do$$

$$Map\ matrix\ initialization, map_{(1:N,1:D)} = 1 \tag{10}$$

$$end$$

$$else$$

$$for\ n\ from\ 1\ to\ N\ do,\ map_{n,randi(D)} = 0$$

$$end$$

$$end$$

Final trial population,

$$TrialPop_{n,d} := \begin{cases} Pop_{n,d} & if \quad map_{n,d} = 1 \\ Mutant_{n,d} & if \quad map_{n,d} = 0 \end{cases} \tag{11}$$

Step 4 (a): Part-II

The final trial population is checked based on the boundary range

$$TrialPop_{n,d} = low_d + rand.(up_d - low_d) \tag{12}$$
$$If\ TrialPop\ (TrialPop_{n,d} < low_d) or (TrialPop_{n,d} > up_d)$$

Step 5: Selection-II

In this stage, the trial population generated in the crossover section is used to verify the objective function. Thereafter, the objective function is recalculated for each population using equation (13) and is compared with the previous population y_{pop} and consequently, the objective function is updated until the maximum iteration number is obtained. Finally, the optimal value of the hyper-parameter is selected using the minimum value of the objective function.

Trial population objective function,

$$y_{TrialPop} = f(Trial) \tag{13}$$
$$Pop_n = TrialPop_n\ if\ y_{n,TrialPop} > y_{n,POp} \tag{14}$$

Gravitational Search Algorithm

Rashedi *et al.* (2009) invented the gravitational search algorithm (GSA) method to achieve an optimal solution in any complex systems. He applied the concept of physics-based algorithms such as the law of gravity and mass interactions to develop GSA. GSA is based on the law of Newtonian gravity and laws of motion. The principle of GSA states that "every particle in the universe attracts every other particle with a force that is directly proportional to their masses and inversely proportional to the square of the distance between them" as expressed in the following equation[2].

$$F = G\frac{M_1 M_2}{R^2} \tag{15}$$

where F denotes the magnitude of the gravitational force; G represents the gravitational constant; M_1 and M_2 characterize the mass of the first and second particles, respectively; and R is the distance between the two particles.

Newton's second law states a relationship between acceleration, $a,$ force, $F,$ and mass, m of a particle which is expressed as follows;

$$a = \frac{F}{m} \tag{16}$$

A new term named Gravitational constant, $G(t)$ was introduced which is assessed using the initial value of the gravitational constant, $G(t_0)$ and the ratio of initial time t_0 and actual time t as follows;

$$G(t) = G(t_0) \times \left(\frac{t_0}{t}\right)^{\beta}\ \beta < 1 \tag{17}$$

The positions of the *N* number of the agents are initialized shown as follows:

$$X_i = \left(X_i^1, \ldots \ldots . X_i^d, \ldots \ldots X_i^n\right), \quad for\ i = 1,2, \ldots, N \qquad (18)$$

where X_{di} is the position of *i-th* agent in the *d-th* dimension and *n* is the space dimension. The mathematical equations for the best and worst value and the masses of each agent are presented as,

$$best(t) = minfit_j(t) \qquad (19)$$
$$Worst(t) = maxfit_j(t) \qquad (20)$$
$$m_i(t) = \frac{fit_i(t) - Worst\ (t)}{best\ (t) - Worst\ (t)} \qquad (21)$$
$$M_i(t) = \frac{m_i(t)}{\sum_{j=1}^{N} m_i(t)} \qquad (22)$$

The total force *F* for the *i-th* agent is evaluated based on the gravitational constant, position, acceleration. Then, the velocity, v and position, x are updated.

$$G(t) = G_0 e^{(-\alpha t/T)} \qquad (23)$$
$$F_{ij}^d(t) = G(t)\frac{M_{pi\times}M_{\alpha j}}{R_{ij} + \varepsilon}\left(X_j^d(t) - X_i^d(t)\right) \qquad (24)$$
$$F_i^d(t) = \sum_{j \in Kbest, j \neq i} rand_j\, F_{ij}^d(t) \qquad (25)$$
$$a_i^d(t) = \frac{F_i^d(t)}{M_i(t)} \qquad (26)$$
$$v_i^d(t+1) = rand_i \times v_i^d(t) + a_i^d(t) \qquad (27)$$
$$x_i^d(t+1) = x_i^d(t) + v_i^d(t+1) \qquad (28)$$

Particle Swarm Optimization

Particle swarm optimization (PSO) is an evolutionary computation technique developed by Eberhart and Kennedey (1995), which is inspired by the social behavior of bird flocking. It is applied by numerous researchers because of its verified robustness, ease of implementation, and global exploration capability in the various application. PSO is a stochastic algorithm which is contingent on the population as a swarm and is used to iteratively find the best results of swam particle with optimal values. The particles in the PSO algorithm are travelling to two locations in the search space. The first location is the best point where the swarm finds the current iteration (local best). The second location is the best point achieved through all previous iterations (global best). The basic principles of the PSO algorithm can be defined in terms of two main factors i.e. velocity of the particle and partial's position in the search space. The velocity of the particle can be expressed through the equation below[3],

$$v_{i,j}^{(t+1)} = w\, v_i^{(t)} + c_1\, r_1\left[pbest_{i,j} - X_{i,j}^{(t)}\right] + c_2 r_2\ \left[gbest_{i,j} - X_{i,j}^{(t)}\right] \qquad (29)$$

where $v_{i,j}^{(t+1)}$ and $v_i^{(t)}$ represent the new velocity and present velocity of the particle, respectively. i is particle number, represented as, $i = [1,2,3, \ldots., N]$ and j is the search space, represented as, $j = [1,2 \ldots, D]$. Acceleration co-efficients are denoted as c_1 and c_1. w is the weight factor. r_1 and r_2 represent the random interval between value (0,1). $pbest$ and $gbest$ denote the best point found through the current iteration (local best) and all previous iterations (global best), respectively.

The positions of the particles in the search space j can be defined as [4].

$$x_{i,j}^{(t+1)}(t+1) = x_{i,j}^{(t)} + v_{i,j}^{(t+1)} \tag{30}$$

where, $x_{i,j}^{(t+1)}$ and $x_{i,j}^{(t)}$ represent the updated swarm position and present swarm position, respectively.

Backpropagation Neural Network Algorithm

A feedforward backpropagation neural network algorithm (BPNN) model consists of three layers; input layer, hidden layer, and output layer, as shown in Fig. 1. The first layer is the input layers to characterize the input variables, the second layer consists of one or more hidden layers and the third layer is the output layer to characterize the output variables. The detail explanation of each step is summarized in the following steps[5]. The flowchart of the BPNN structure is divided into four steps as shown in Fig. 2.

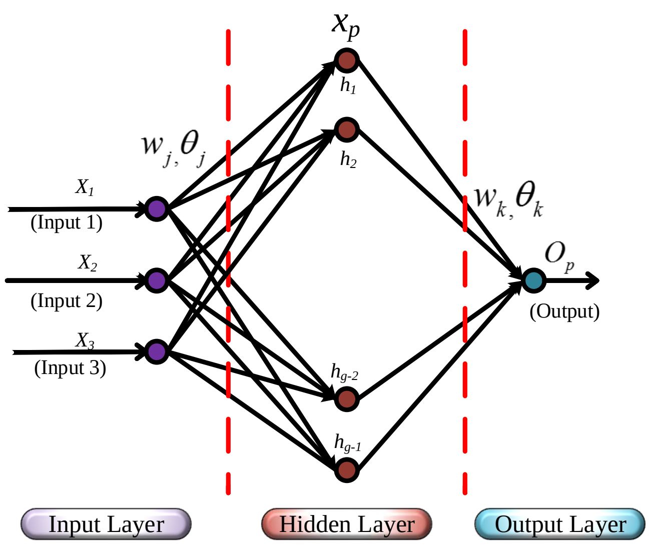

Fig 1. Structure of BPNN for SOC estimation

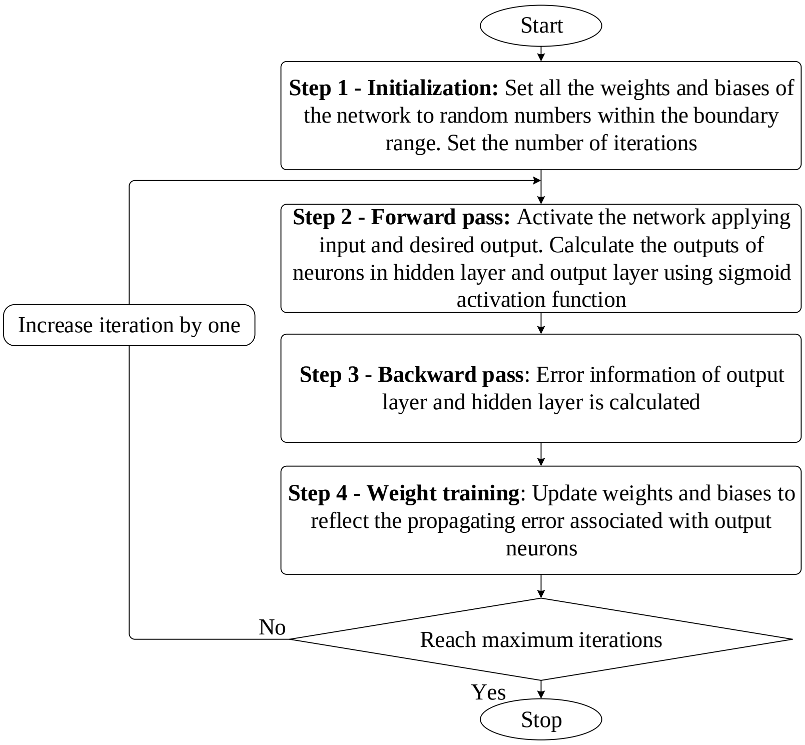

Fig 2. Flowchart of BPNN model

Step 1: Initialize weight and bias to random variables.

Step 2: For input pattern *p*, the *i-th* input layer node holds $x_{p,i}$. Net input to *j-th* node in the hidden layer is

$$net_j = \sum_j w_{i,j} x_i + \theta_{i,j} \tag{31}$$

where, $w_{i,j}$ is the weight from the input layer to hidden layer, $\theta_{i,j}$ represents the bias from the input layer to the hidden layer.

The output of *j-th* node in the hidden layer is

$$O_j = f(net_j) \tag{32}$$

The hidden layer uses the log-sigmoid function as a transfer function which is defined as

$$f(net) = \frac{1}{1 + e^{(-net)}} \tag{34}$$

Net input to *k-th* node in the output layer is

$$net_k = \sum_k w_{j,k} O_j + \theta_{j,k} \tag{35}$$

$w_{k,j}, \theta_{k,j,}$ are the weight and bias from the hidden layer to the output layer. Linear activation function is used in the output layer. Output of *k-th* node in output layer is,

$$O_k = f(net_k) \tag{36}$$

Step 3: The error is estimated and propagates backward from the output layer to the hidden layer. The error in the output layer is computed as

$$e_k = T_k - O_k \tag{37}$$

$$\partial_k = e_k\, f'(net_k) \tag{38}$$

T_k is the true output

The error in the hidden layer is calculated as

$$\partial_j = f'\left(net_j\right) \partial_k w_{j,k} \tag{39}$$

Step 4: In this stage, weights and biases are updated.

Weights are updated using the following equations

$$\Delta w_{j,k} = \alpha \partial_k O_j \tag{40}$$

$$w_{j,k} = w_{j,k} + \Delta w_{j,k} \tag{41}$$

$$\Delta w_{i,j} = \alpha \partial_j x_i \tag{42}$$

$$w_{i,j} = w_{i,j} + \Delta w_{i,j} \tag{43}$$

where α is the learning rate.

Biases are updated using the following equations,

$$\Delta \theta_{j,k} = \alpha \partial_k \tag{44}$$

$$\theta_{j,k} = \theta_{j,k} + \Delta \theta_{j,k} \tag{45}$$

$$\Delta \theta_{i,j} = \alpha \partial_j \tag{46}$$

$$\theta_{i,j} = \theta_{i,j} + \Delta \theta_{i,j} \tag{47}$$

Radial Basis Function Neural Network Algorithm

Radial basis function neural network (RBFNN) is a feed forward self-learning algorithm which consists of a non-linear function with a symmetrical organization. RBFNN has good global approximation performance [6].

The structure of RBFNN consists of three-layer including one input layer, one hidden layer and one output layer, as shown in Fig. 3.

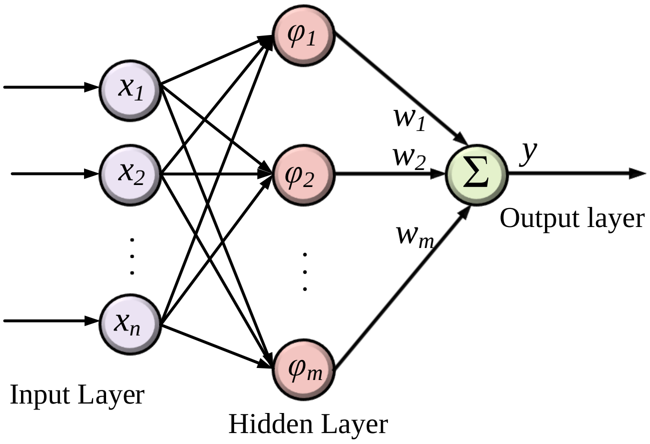

Fig **Error! No text of specified style in document.**. The structure of RBFNN for SOC estimation

In this research, the Gaussian distribution is used as an activation function to estimate SOC. RBFNN has hidden neurons in the hidden layer neurons which are called RBF units. The location of the Gaussian function is characterized by two key parameters of RBF units named center and width. The center and width terms of the *j* Gaussian distribution function are denoted by λ_m and σ_m, respectively. The output of the m_{th} hidden neuron of the RBFNN can be expressed by[6],

$$\phi_m(n) = \phi_m\{x(n), \lambda_m(n), \sigma_m(n)\} \quad (48)$$

$$= e^{-\frac{\|x(n)-\lambda_m(n)\|^2}{\sigma_m^2(n)}, for\ m=1,2,\ldots,M} \quad (49)$$

where, x is the input vector in the input layer. The output of RBFNN comprises linear function and is calculated by multiplying the weight values with hidden nodes which is shown in the following equation,

$$y_k = \sum_{m=1}^{M} w_{km}\phi_m(n), for\ k = 1,2,\ldots,m \quad (50)$$

where, y_k represents the output of the k_{th} neuron in the output layer, w_{km} denotes the weight, connecting the m_{th} hidden neurons to the k_{th} output layer neuron and ϕ_m is the hidden layer output for m_{th} neurons.

Extreme Learning Machine Algorithm

Extreme learning machine algorithm (ELM) is appropriate for predicting outcomes in complex and nonlinear systems. ELM has a number of advantageous features such as better scalability, better generalization performance for regression and classification, better approximation of any target continuous function, lower computation complexity and faster learning speed which help to deliver better estimation results than other machine learning algorithms[7]. ELM is designed using three layers, one input layer, one hidden layer, and one output layer, as depicted in Fig. 4. The execution of ELM is performed by randomly assigning the input weights and hidden layers biases. The steps of ELM are described as follows[8]

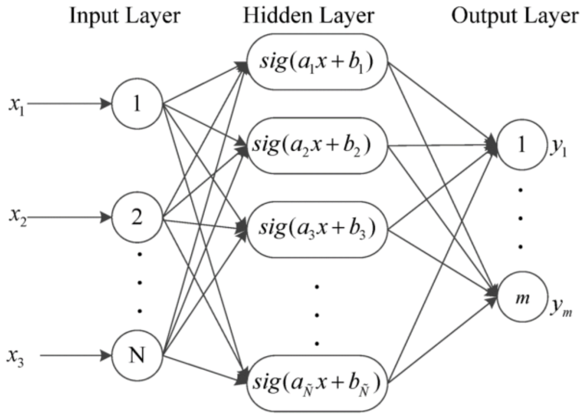

Fig 4. Single layer ELM model structure

i. At first, the parameters are assigned randomly. The input weight vector and hidden layer bias are represented as $x_i = [x_{i1,}x_{i2,} \dots \dots x_{iN,}]^T$ and b_i respectively where i is the number of neurons in hidden layer. The hidden neurons are assigned as $\tilde{N}$. The value of $\tilde{N}$ can be changed in order to achieve reasonable accuracy.

ii. Calculate the output matrix of the output layer. The mathematical expression is represented by,

$$\sum_{i=1}^{\tilde{N}} \beta_i f_i(x_i) = \sum_{i=1}^{\tilde{N}} \beta_i f(a_i.x_j + b_j) = t_{j,}\ j = 1, \dots, N \tag{51}$$

Where $a_i = [a_{i1,}a_{i2}, \dots, a_{in}]^T$ represents the weight vector which connects the input nodes and *i-th* hidden nodes. $\beta_i = [\beta_{i1,}\beta_{i2}, \dots, \beta_{in}]^T$ represents the output weight which connect the *i-th* hidden layer neuron and output layer neuron. *f ()* is the activation function which is determined before training. In this research, the most popular sigmoid function is used as the activation function [9].

$$f(a_i.x_j + b_j) = \frac{1}{1+e^{-(a_i.x_j+b_j)}}^{-1}, i = 1, \dots., L, j = 1, \dots., N \tag{52}$$

Equation (51) can be represented compactly as,

$$H\beta = T \tag{53}$$

Where $H(a_1, \dots, a_{\tilde{N},}b_1, \dots, b_{\tilde{N},,}x_1, \dots, x_N)$

$$H = \begin{bmatrix} f(a_1.x_1 + b_1) & \dots & f(a_{\tilde{N},}.x_1 + b_{\tilde{N},}) \\ \dots & \dots & \dots \\ f(a_1.x_N + b_1) & \dots & f(a_{\tilde{N},}.x_N + b_{\tilde{N},}) \end{bmatrix}_{N \times N}$$

$$\beta = \begin{bmatrix} \beta_1^T \\ . \\ \vdots \\ \beta_N^T \end{bmatrix}_{N \times m} \quad T = \begin{bmatrix} t_1^T \\ . \\ \vdots \\ T_N^T \end{bmatrix}_{N \times m}$$

H is the matrix of the hidden layer of the ELM network.

iii. The hidden layer output matrix *H* is determined by randomly allocated input weights and hidden layer biases. Hence, a linear equation $H\beta = T$ is obtained.

$$\left\| H\left(a_1, \dots, a_{\tilde{N},} b_1, \dots, b_{\tilde{N},}\right)\hat{\beta} - T \right\| = \min_{\beta} \left\| H\left(a_1, \dots, a_{\tilde{N},} b_1, \dots, b_{\tilde{N},}\right)\hat{\beta} - T \right\| \quad (54)$$

The least square solution is used to solve the above equation. The output weight β is estimated by,

$$\hat{\beta} = H^{+}T \quad (55)$$

where H^{+} is the Moore–Penrose generalized inverse of H. The optimal solution $\hat{\beta}$ features the lower training error and optimal generalization performance.

Deep Recurrent Neural Network Algorithm

Deep recurrent neural network algorithm (DRNN) is predominantly well preferred for the prediction of complex time series problem due to its powerful computational tool. The DRNN is successfully implemented for parameter projecting in numerous application such as industries, image processing and forecasting [10]. Moreover, DRNN comprises a unique dynamic memory, through which complex system can be addressed with the appropriate value of weights. The conventional recurrent neural network does not hold more than two non-linear functions in the hidden layer [11]. With the facility of real-world data availability and enhancement of computing power and memory storage system, the deeper architecture of recurrent neural network has been explored in many application. The learning procedure of the DRNN is implemented through one of the two ways such as feed-forward connection and feedback connection [12]. Although the training process of DRNN has some similarity with the feed forward neural network, there are some differences between the two processes. The output response is evaluated based on a repeated feedback process which contains the hidden output of that particular instance and hidden output from the previous instance. The information is stored on the feedback loop of the previous phase and final output is predicted based on the instantaneous output and the previous output. The basic structure of the DRNN is presented in Fig. 5.

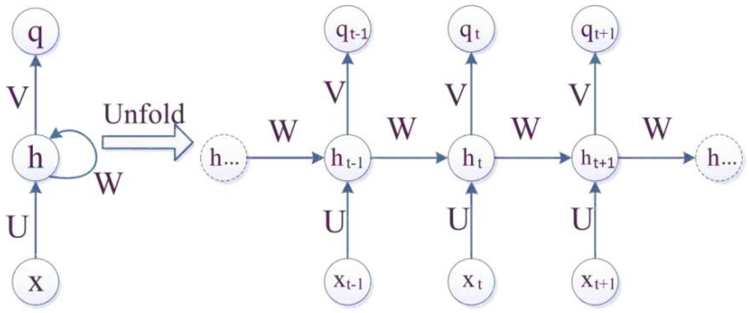

Fig. 5. Structure of DRNN for SOC estimation

The DRNN is used to estimate SOC at time t with input series ($L = L_{1,} \dots L_t$), hidden vector series ($P = P_{1,} \dots P_t$) and output vector series ($y = y_{1,} \dots y_t$). The equations are shown below,

$$net_l = (W_{hL}L_t + W_{PP}P_{t-1} + b_P) \quad (56)$$

$$P_t = f(net_l) \quad (57)$$

$$net_t = \left(W_{yh}P_t + b_y\right) \quad (58)$$

$$y_t = f(net_t) \quad (59)$$

where W_{hL} is the weight between the input layer and the hidden layer, W_{PP} is the weight between a hidden layer and itself at adjacent time steps, W_{yh} is the weight between the hidden layer and output layer. The hidden layer

bias and output layer bias are represented by b_P and b_y. *f*() denotes the sigmoid activation function. P_t and y_t represent the output of hidden layer and output layer respectively.

Random Forest Algorithm

Breiman (2001) introduced an enhanced machine leaning algorithm named random forest (RF). RF does not overfit as a predictor, runs fast and efficiently when handling large datasets, thus leading to superior performance. RF is based on set on predictors which depend on trees in the forests through the random values of each tree. The RF is modeled by picking up a group of small input dataset and then splitting them in a random order. The procedures of RF begin with the formation of new dataset equal to the length of the original data. The bootstrapping technique is used to choose the data in a random way from the original data set. A sequence of binary splits is formed from the new dataset to create the decision trees. The responses to the estimated data are created using classification and regression. The response comes from the decision of each tree in the forest that generates from the root node and then transfers to a leaf node [14]. The structure of RF algorithm is shown in Fig. 6.

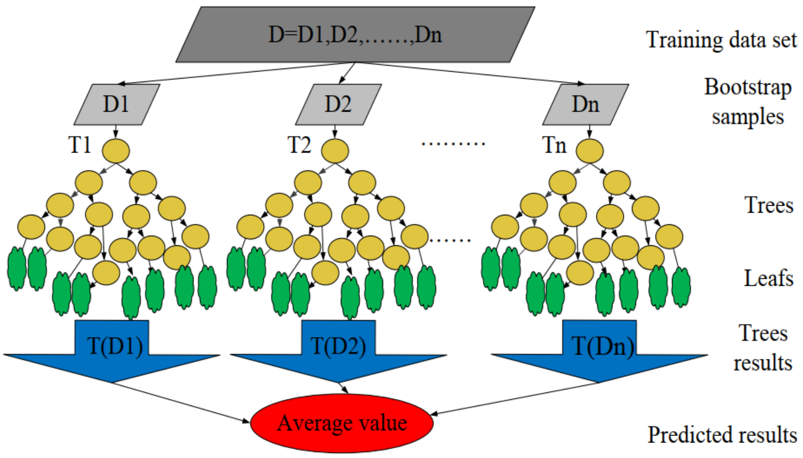

Fig. 6. Flow diagram of random forest algorithm structure

The RF regression is developed using the p dimension input vector of $X = x_1, x_2, \ldots \ldots, x_p$ to build the forest. A set of K tress $\{T_1(x), T_2(x), \ldots, T_k(x)\}$ is used inside the forest. The actual output value is determined by each tree, represented as $\hat{Y}_1 = T_1(X), \ldots, \hat{Y}_m = T_1(X)$, where $m = 1, \ldots, K$. The outcome of RF is evaluated by estimating the average of all trees predictors, as expressed in the following equation,

$$Predict_{RF}(X) = \frac{1}{k}\sum_{k=1}^{K} \widehat{Y_N}(X) \qquad (60)$$

The training dataset $D = D_{1,} D_{2,} \ldots, D_{n,} = \{(x_1, y_1), (x_2, y_2), \ldots, (x_n, y_n)\}$ is drawn independently from input and output where $x_{i,} i = 1, \ldots, n$ denotes the input vector training dataset and $y_{i,} i = 1, \ldots, n$ expresses the output vector training dataset. The training procedures for the growth of tree in the forest are explained as follows

i. A bootstrap sample is drawn for each regression tree through the available training dataset. A bootstrap sample is a random sample conducted with replacement. A different subset of dataset is employed to improve the tree model for each bootstrap sample. The Out-of-bag (OBB) samples are formed by leaving one third of the dataset. A total of two third sample is available of the new training sample. OBB is the process of neglecting values from each bootstrap sample. OBB data plays a key part in tree development and is checked with the estimated values at each step.

ii. At each node of the regression tree in the bootstrap sample, the best split is chosen among the randomly selected subset. The node in each tree plays a vital role in the correction of changing parameters of the algorithm.

iii. Each tree is designed to the largest extended possible without pruning.

iv. The predictions are evaluated by placing each OBB observations of the test data for each tree. The mean value of predictions of the total regression trees are calculated through equation (60).

The accuracy and error rate of RF are evaluated though the minimization of the OBB. The OBB error is an important feature of RF. As mentioned earlier, each tree is developed based on the bootstrap sample that consists of roughly two thirds of the training data. The remaining one-third (OBB) of the training data is not included in the learning sample for this tree and can be used for testing. The MSE is employed to observe the OBB error which is found by assessing the deviation between predicted and reference values, as shown the following equation,

$$MSE \approx MSE^{OBB} = \frac{1}{n}\sum_{i=1}^{n}\left(\hat{Y}(X_i) - Y_i\right)^2 \tag{61}$$

where $\hat{Y}(X_i)$ represents the predicted output, Y_i represents the observed output and n is the total number of samples.

Reference

1. Civicioglu, P. Backtracking Search Optimization Algorithm for numerical optimization problems. *Appl. Math. Comput.* **219,** 8121–8144 (2013).
2. Rashedi, E., Nezamabadi-pour, H. & Saryazdi, S. GSA: A Gravitational Search Algorithm. *Inf. Sci. (Ny).* **179,** 2232–2248 (2009).
3. Hossain Lipu, M. S., Hannan, M. A., Hussain, A. & Saad, M. H. M. Optimal BP neural network algorithm for state of charge estimation of lithium-ion battery using PSO with PCA feature selection. *J. Renew. Sustain. Energy* **9,** (2017).
4. Latha, K., Rajinikanth, V. & Surekha, P. M. PSO-Based PID Controller Design for a Class of Stable and Unstable Systems. *ISRN Artif. Intell.* **2013,** 1–11 (2013).
5. Hannan, M. A., Lipu, M. S. H., Hussain, A., Saad, M. H. & Ayob, A. Neural Network Approach for Estimating State of Charge of Lithium-ion Battery Using Backtracking Search Algorithm. *IEEE Access* **6,** 10069–10079 (2018).
6. Chang, W.-Y. Estimation of the state of charge for a LFP battery using a hybrid method that combines a RBF neural network, an OLS algorithm and AGA. *Int. J. Electr. Power Energy Syst.* **53,** 603–611 (2013).
7. Hussain Lipu, M. S. *et al.* Extreme Learning Machine Model for State of Charge Estimation of Lithium-ion battery Using Gravitational Search Algorithm. *IEEE Trans. Ind. Appl.* **55,** 4225–4234 (2019).
8. Sattar, A. M. A., Ertuğrul, Ö. F., Gharabaghi, B., McBean, E. A. & Cao, J. Extreme learning machine model for water network management. *Neural Comput. Appl.* 1–13 (2017).
9. Guang-Bin Huang, G. Bin. Learning capability and storage capacity of two-hidden-layer feedforward networks. *IEEE Trans. Neural Networks* **14,** 274–281 (2003).
10. Chuan-long, Y., Yue-fei, Z., Jin-long, F. & Xin-zheng, H. A Deep Learning Approach for Intrusion Detection using Recurrent Neural Networks. *IEEE Access* **5,** 21954–21961 (2017).
11. Chemali, E., Kollmeyer, P. J., Preindl, M. & Emadi, A. State-of-charge estimation of Li-ion batteries using deep neural networks: A machine learning approach. *J. Power Sources* **400,** 242–255 (2018).
12. Hinton, G. *et al.* Deep Neural Networks for Acoustic Modeling in Speech Recognition: The Shared Views of Four Research Groups. *IEEE Signal Process. Mag.* **29,** 82–97 (2012).
13. Breiman, L. Random Forests. *Mach. Learn.* **45,** 5–32 (2001).
14. Ibrahim, I. A. & Khatib, T. A novel hybrid model for hourly global solar radiation prediction using random forests technique and firefly algorithm. *Energy Convers. Manag.* **138,** 413–425 (2017).
